# Costs of and Investment in Mate-Guarding in Wild Long-Tailed Macaques (*Macaca fascicularis*): Influences of Female Characteristics and Male–Female Social Bonds

**DOI:** 10.1007/s10764-014-9775-3

**Published:** 2014-05-07

**Authors:** Cédric Girard-Buttoz, Michael Heistermann, Erdiansyah Rahmi, Muhammad Agil, Panji Ahmad Fauzan, Antje Engelhardt

**Affiliations:** 1 Jr. Research Group Primate Sexual Selection, German Primate Centre, 37077 Göttingen, Germany; 2 Courant Research Centre Evolution of Social Behaviour, Georg-August University, 37077 Göttingen, Germany; 3 Endocrinology Laboratory, German Primate Centre, 37077 Göttingen, Germany; 4 Faculty of Veterinary Medicine, Syiah Kuala University, Banda Aceh, Indonesia; 5 Faculty of Veterinary Medicine, Bogor Agricultural University, Bogor, Indonesia

**Keywords:** Feeding costs, Glucocorticoids, *Macaca fascicularis*, Mate choice, Reproductive effort, Vigilance

## Abstract

Male primates living in multimale groups tend to direct mate and mate-guarding choices toward females of high reproductive value, i.e., high-ranking, parous females, or females with which they share strong bonds. Little is known, however, about the constraints that may limit male mate-guarding choices (the costs of this behavior) and the influence of the females’ quality on male investment in mate-guarding. We aimed to study the effects of female rank, parity status, and male–female social bond strength on the costs of and investment in mate-guarding by males. We carried out our study during two reproductive seasons on three groups of wild long-tailed macaques in Indonesia. We combined behavioral observations on male locomotion and activity with noninvasive measurements of fecal glucocorticoids (fGC). Males spent less time feeding when mate-guarding nulliparous females than when mate-guarding parous females and tended to have higher fGC levels when mate-guarding low-ranking nulliparous females than when mate-guarding high-ranking nulliparous ones. Evolution should thus favor male choice for high-ranking parous females because such a decision brings benefits at proximate (reduced costs of mate-guarding) and ultimate (higher reproductive value) levels. Further, male investment in mate-guarding was flexible and contingent on female reproductive and social value. Males were more vigilant and more aggressive toward other males when mate-guarding females to which they were strongly bonded and/or high-ranking ones than when mate-guarding other females. Our findings bring a new dimension to the study of mate choice by showing that males not only mate preferentially with high-quality females but may also aim to secure paternity with these females through optimized monopolization.

## Introduction

Studies of mate choice traditionally focus on females because these are usually the sex that invests the most into reproduction and, hence, should be more selective in regard to their mating partners (Andersson 1994; Trivers 1972). Variability in the quality of available females and the costs of mating may, however, favor the evolution of mate choice in males as well, even in species with no sex-role reversal (Edward and Chapman 2011; Kokko and Monaghan 2001). In particular, male mate choice is particularly likely to evolve when multiple females are available at the same time and when the rate of encounters with females is high (Kokko and Monaghan 2001), such as in many group-living primates.

Several primate species have evolved a certain degree of male mate choice to limit the costs of reproducing and allocate limited sperm resources toward the most valuable females (Kappeler 2012; Keddy-Hector 1992; Setchell and Kappeler 2003). Male–male competition for access to mates, including monopolization of females, is often costly for male primates because it may increase the risk of injury (Drews 1996) and affect a male’s feeding time, energy balance, or physiological stress levels (Alberts *et al*. 1996; Bergman *et al*. 2005; Georgiev 2012; Girard-Buttoz 2014; Girard-Buttoz *et al*. 2014). In addition, successive ejaculations may impair sperm performance (Marson *et al*. 1989). Sperm is, therefore, a limited resource (Wallen 2001) and males may face a trade-off between current and future mating opportunities when several females are receptive simultaneously (Kappeler 2012). Males should, therefore, choose to compete for and mate with those females most likely to be fertile and to produce offspring surviving until the next generation (Setchell and Kappeler 2003). Indeed, males of several species have been observed to concentrate their mating effort on females during their conceptive cycles, e.g., chimpanzees (*Pan troglodytes schweinfurthii*: Emery Thompson and Wrangham 2008) and chacma baboons (*Papio hamadryas ursinus*: Weingrill *et al*. 2003) and to preferentially mate with high-ranking females, e.g., Barbary macaques (*Macaca sylvanus*: Kuester and Paul 1996) and long-tailed macaques (*M. fascicularis*: Berenstain and Wade 1983; de Ruiter *et al*. 1994). These females produce offspring of better quality than low-ranking females, i.e., offspring more likely to survive until adulthood and to achieve a high rank position in the future (Majolo *et al*. 2012; Robbins *et al*. 2011; Setchell *et al*. 2002; van Noordwijk and van Schaik 1999, 2001). For similar reasons, males may mate more frequently with “experienced” parous females that have already produced and successfully raised an infant than with nulliparous females, e.g., chimpanzees (Muller *et al*. 2006), mandrills *Mandrillus sphinx*: Setchell 1999), savannah baboons (*Papio cynocephalus*: Smuts 1985). Finally, in some species, males exhibit mating preferences toward females with which they have strong social bonds, independently of female rank, parity, or fertility status, e.g., rhesus macaques (*Macaca mulatta*: Chapais 1983), Japanese macaques (*M. fuscata*: Takahata 1982), and savannah baboons (Smuts 1985).

Being a selective male may make sense particularly in species in which males engage in costly female monopolization, i.e., mate-guarding, over an extended period of time to secure paternity (Manson 1997). While mate-guarding increases the mating or reproductive success of male primates significantly (rhesus macaques: Berard *et al*. 1994; Bercovitch 1997; long-tailed macaques: de Ruiter *et al*. 1994; Engelhardt *et al*. 2006; Japanese macaques: Matsubara 2003; mandrills: Setchell *et al*. 2005), this behavior also entails costs, in at least some species. In a number of baboon and macaque species, mate-guarding led to a reduction in male feeding time (Alberts *et al*. 1996; Girard-Buttoz *et al*. 2014; Matsubara 2003; Packer 1979; Rasmussen 1985). Males may also face physiological constraints during mate-guarding. Male long-tailed macaques and chacma baboons have higher fecal glucocorticoid levels (a marker of physiological stress) when mate-guarding than when not mate-guarding females (Bergman *et al*. 2005; Girard-Buttoz 2014). These costs may, however, vary depending on which female is mate-guarded. Given that high-ranking and/or parous females may be of higher reproductive value for males than low-ranking and/or nulliparous ones, males mate-guarding high-ranking and/or parous females may face higher costs than when mate-guarding other females because of the increased challenges by other males. At the same time, high-ranking females may travel shorter distances than low-ranking females, have priority of access to high-quality food, and face less risk of predation by spending more time in the core of the group than low-ranking females, as is the case in baboons and macaques for example (Ron *et al*. 1996; Saito 1996; van Noordwijk and van Schaik 1987; Vogel 2005). Males mate-guarding these females may, thus, face reduced costs when adjusting their activity, locomotion, and spatial positioning to the guarded female. The direction of the relationship between female reproductive value and costs of mate-guarding for males therefore remains unclear.

In addition to female reproductive value, male–female social bonds are also likely to influence the costs of mate-guarding. On the one hand, these bonds can affect female cooperation during mate-guarding, e.g., yellow baboons (Rasmussen 1980), which may, in turn, reduce the costs of monopolization. On the other hand, males may invest more energy and mate-guard females with which they are strongly bonded more thoroughly than other females in order to maintain the fitness benefit males may derive from long-term male–female social bond. For example, in rhesus macaques, the strength of these bonds is positively related to a male’s reproductive success (Kulik *et al*. 2012; Massen *et al*. 2012).

In summary, previous studies suggest that female reproductive value and male–female social bond strength may either increase or decrease the costs of mate-guarding, depending on the factors at play. However, these relationships have no yet been tested. It is, therefore, difficult to predict the direction of the relationship between female identity and costs of mate-guarding, and more empirical studies addressing this question directly are needed.

We aimed to quantify the influence of female rank and parity status and male–female social bonds on the costs of, and investment in, mate-guarding in male long-tailed macaques. Male reproductive success is highly skewed toward the α male in this species (de Ruiter *et al*. 1994; Engelhardt *et al*. 2006). However, high-ranked males mate-guard females to a lower extent than predicted by the Priority of Access model (Altmann 1962; Engelhardt *et al*. 2006). Because males are able to discern a female’s fertile phase (Engelhardt *et al*. 2004), we suggest that this lower than expected degree of α male monopoly derives from behavioral or social constraints associated with the costs of mate-guarding (Girard-Buttoz 2014). In previous studies, we found that mate-guarding influences male behavior and physiology in long-tailed macaques. Males spent less time feeding, climbed less distance, received more aggression, were more vigilant and exhibited higher levels of stress hormones while mate-guarding females than when not (Girard-Buttoz 2014; Girard-Buttoz *et al*. 2014). Male energetic status (assessed through urinary C-peptide measures; Girard-Buttoz *et al*. 2011) was, however, not affected by mate-guarding (Girard-Buttoz *et al*. 2014). Although we documented some costs of mate-guarding in long-tailed macaques, the extent to which these costs varied across the guarded females remains unclear. In this species, top-ranking males concentrate their mate-guarding effort on high-ranking and parous females (de Ruiter *et al*. 1994; Engelhardt *et al*. 2006), as these produce better quality offspring (van Noordwijk and van Schaik 1987). The extent to which this choice is based on differences in the costs during mate-guarding remains, however, unclear.

We first quantified the effect of female value and male–female social bonds on behavioral and physiological parameters that have been shown to be affected by mate-guarding, i.e., feeding time, climbing distance, and physiological stress levels (Girard-Buttoz 2014; Girard-Buttoz *et al*. 2014). Second, we examined whether males were more vigilant and more aggressive or kept a closer distance to the female when mate-guarding females of high reproductive value or with whom they were closely bonded than when mate-guarding other females.

## Methods

### Animals and Study Site

We carried out the study on three groups of wild long-tailed macaques (*Macaca fascicularis*) living in the primary lowland rain forest surrounding the Ketambe research Station (3°41′N, 97°39′E), Gunung Leuser National Park, North Sumatra, Indonesia. The forest structure and phenological composition has been described in detail by Rijksen (1978) and van Schaik and Mirmanto (1985). The long-tailed macaques in the area have been studied since 1979 (de Ruiter *et al*. 1994; Engelhardt *et al*. 2004; van Schaik and van Noordwijk 1985). For our study, we focused on three groups: Camp (C), Ketambe Bawa (KB), and Ketambe Atas (KA). We collected fecal samples and behavioral data during two consecutive mating periods extending from March to July 2010 and from December 2010 to April 2011. We defined a mating period as the period between the first mate-guarding day and the last mate-guarding day observed in any of the three groups, by any male (see later for definition of mate-guarding). All adult individuals were individually known and well habituated to human observers. The total size of the social groups varied from 22 to 58 individuals (Table I; for details see Girard-Buttoz *et al*. 2014). Between January and April 2011, four males traveled back and forth between the groups KA and KB and associated with one of the groups for periods between a few hours up to 3 wk before traveling back to the other group.

**Table I:** Observation time on mate-guarding (MG) days, number of faecal samples measured and characteristics of the guarded females for each of the study male long-tailed macaques (*Macaca fascicularis*) at Ketambe, Gunung Leuser National Park, Indonesia (2010-2011).

Group	Camp		Ketambe Atas		Ketambe Bawa	
Male rank	α	β	α	β	α	Β
Number of mating periods	2	2	1	1	2	2
Focal observation time on MG days (hours)	253.6	73.6	118.4	75.3	160.7	51.6
Number of MG days of observation	47	15	39	31	45	15
Number of faecal samples	32	6	24	16	26	9
Number of adult males in the group	6–9	6–9	4–7	4–7	4–8	4–8
Number of adult females in the group	14–15	14–15	7	7	9–10	9–10
Number of females mate-guarded	6	8	4	3	7	6
Number of nulliparous females guarded	1	1	1	0	5	4
Range of guarded female ranks	1–15	4–15	1–7	1–4	1–9	1–9

We conducted the study completely noninvasively and under the permission of the authorities of Indonesia. We adhered to the Guidelines of the Use of Animals in Research, the legal requirements of Indonesia, and the guidelines of the involved institutes.

### Behavioral Data Collection

C. Girard-Buttoz and six experienced Indonesian and international field assistants collected the behavioral data. C. Girard-Buttoz trained all assistants and we assessed interobserver reliability repeatedly (measurement of agreement κ > 0.8 for each assistant and for all behaviors). The observations covered two mating periods for two groups (C and KB) and one mating period for the third group (KA). From March to July 2010, four observers followed groups C and KB every day and from December 2010 until April 2011 we followed all three groups generally every other day and frequency of observations increased to every day when α and/or β males were observed mate-guarding. Each day, we followed the groups from dawn to dusk. We focused our behavioral observations on α and β males because they are known to mate-guard females most extensively (Engelhardt *et al*. 2006). The α and β males of each group were the focal individuals for half or entire days depending on the number of observers available.

We recorded the activity of the focal individual every minute using instantaneous sampling (Altmann 1974) comprising the following categories: resting, being vigilant (monitoring the surrounding environment by looking in different directions, being either still or moving, and while not involved in feeding or social activity), feeding (handling and consuming food), drinking, traveling (continuous locomotion during ≥1 min with no foraging activity and no social interactions), aggressing, affiliating (including copulation), grooming, self-grooming. We also recorded every minute the canopy height (six categories: 0: focal individual on the ground, 1: 1–5 m; 2: 5–10 m; 3: 10–15 m; 4: 15–20 m; 5: 20–25 m; 6: >25 m), the mate-guarding behavior of the focal male and the distance between him and the mate-guarded female. Whether a male was mate-guarding a female or not on a given minute was coded *a posteriori*. We considered that a male was “mate-guarding” when he followed a sexually active female for more than 5 consecutive minutes and maintained a distance of ≤10 m to the female. We considered that a female was sexually active if she was observed copulating at least once on a given day. If the female moved away from the male and the male did not follow her for >2 min we considered the mate-guarding activity to have ended. “Mate-guarding days” refers to the days during which the male mate-guarded females for ≥25 % of the observation time. In addition, we recorded all copulations and aggressions (including submissive expressions) between any adult individuals (all occurrence sampling for the focal male and *ad libitum* for all the other individuals). Aggressions comprised threatening, chasing, hitting, and biting. We also recorded all occurrence of approaches (defined as one individual entering within a 1 m radius of another individual) between the focal male and any other adult individual in the group. Finally, we recorded the identities of all adult males within 10 m of the focal individual every 5 min.

### Vertical Traveling Distance

To calculate the vertical distance traveled, we used the centre of each height category as an estimate for the height at which the male was at each minute-scan-point, e.g., 7.5 m for category 2 or 12.5 m for category 3, and calculated the height difference between consecutive minute-scans.

### Determination of Female Dominance Hierarchy and Parity Status

During the focal samples, we recorded *ad libitum* (Altmann 1974) any agonistic interaction and the occurrence of “bared-teeth face,” a unidirectional submissive display (van Hooff 1967) between any adult members of the groups. We built the dominance hierarchy for females based on 488 dyadic aggressive or submissive interactions (312, 132, and 64 in groups C, KA, and KB, respectively) in which a clear winner and loser could be identified. The aggressive interactions used in this analysis were chase and displacement. We then entered winner and loser into a sociometric matrix and compiled dominance ranks with Matman 1.1.4 using the I&SI method with 10,000 randomizations (de Vries 1998). The percentages of unknown relationships were 32.4%, 19.1%, and 33.3% in group C, KA, and KB, respectively. We used I&SI because this method performs better than the David’s score when interactions between some dyads are missing (Neumann *et al*. 2011). Landau’s corrected linearity indexes were 0.54, 0.89, and 0.74 in group C, KA, and KB, respectively and the hierarchy was significantly linear in each of the three groups (all *P* < 0.02). Before statistical analysis we standardized female ordinal rank to a mean of 0 and a standard deviation of 1 in each group to obtain a range of values comparable between the three groups containing different number of females (Table I). After standardization, high-ranking females received a scores that were <0 and low-ranking females a score that were >0.

Because the long-tailed macaques in the Ketambe research area have not been studied between 2001 and the start of our study in 2010 we did not know the reproductive history of the focal females. Consequently, we assessed the parity status of the female visually based on the size of the nipples. In long-tailed macaques nulliparous female nipples are similar to male nipples and distinctively shorter than parous female nipples.

### Determination of Male–Female Social Bond

We measured dyadic male–female social bond strength using an approach inspired from the calculation of the “composite index of sociality” (Silk *et al*. 2006). However, because we did not collect focal behavioral observations on all the males present in each of the groups, we could not compute a “composite index of sociality.” Instead, we used the number of approaches and grooming time to calculate a “male-centered” association index (hereafter AI) between males and females. We computed the AI for each male–female dyad as follows: AI = [(*G*_ij_/*G*_ix_) + (*A*_ij_/*A*_ix_)]/2 where *G*_ij_/*G*_ix_ is the grooming time of male i with female j (*G*_ij_) relative to the total grooming time of male i with all females in the group (*G*_ix_). Similarly, *A*_ij_/*A*_ix_ is the number of time male i approached or was approached by female j (*A*_ij_), relative to the total number of time male i was approached or approached all females in the group (*A*_ij_). To obtain a measure independent of male mate-guarding activity, we used only data collected on days during which the male did not mate-guard females at all. The mean focal observation time on non–mate-guarding days was 160.2 h per male (range: 39.6–265.4 h).

### Determination of Fruit Availability

Fruit availability is known to affect feeding time, distance climbed, and glucocorticoid levels in male long-tailed macaques (Girard-Buttoz 2014; Girard-Buttoz *et al*. 2014). It was therefore important to control for this parameter in our analysis and we monitored fruit trees to assess fruit availability. In each of the three groups studied, we randomly selected 40 locations, covering the entire home ranges (120 locations in total over the three home ranges). At each location, we randomly selected three trees from three different species among the tree species producing fruit eaten by long-tailed macaques (Ungar 1995). In total we selected 360 trees, from 87 different species (120 trees for each group’s home range). A field assistant experienced in phenology surveyed each tree monthly, within the last 3 d of every month, and recorded fruit abundance using a logarithmic scale (0: absence, 1: 1–10 items, 2: 11–100, 3: 101–1000, 4:1001–10,000, 5: >10,000). For the analyses, we used percentage of trees fruiting as an index of fruit availability. This index was highly correlated to the mean monthly score of fruit abundance in each territory (Spearman signed rank test, *N* = 36, *S* = 720, *r =* 0.91, *P* < 0.001). The percentage of trees fruiting varied between 6.8 and 30.9%.

### Fecal Sample Collection and Hormone Analysis

During mate-guarding periods, we collected fecal samples every third day from the mate-guarding males. Immediately after defecation, we homogenized samples, collected 2–3 g of feces, stored them in a polypropylene vial, and placed them on ice in a thermos bottle. At the end of each fieldwork day, the samples were frozen at –20°C in a freezer. In July 2011, we transported all samples, on ice, to the hormone laboratory of the Bogor Agricultural University (IPB) and then freeze-dried and pulverized them before transportation to the Endocrinology Laboratory of the German Primate Centre for fecal glucocorticoid (fGC) analysis.

For hormone analysis, we extracted an aliquot (50–70 mg) of the fecal powder within 3 ml of 80% methanol by vortex mixing for 10 min (Heistermann *et al*. 1995). To monitor changes in fGC levels, we analyzed fecal extracts for immunoreactive 11ß-hydroxyetiocholanolone (3α,11ß-dihydroxy-CM), a group-specific measurement of 5-reduced 3α,11ß-dihydroxylated cortisol metabolites (Ganswindt *et al*. 2003; Möstl and Palme 2002). The assay has been previously validated for assessing adrenocortical activity from feces in long-tailed macaques (Heistermann *et al*. 2006). We carried out hormone measurements by microtiter plate enzymeimmunoassay according to methods previously described (Ganswindt *et al*. 2003; Girard-Buttoz *et al*. 2009). Intra- and interassay coefficients of variation of high- and low-value quality controls were 8.9% and 9.9% (high) and 6.3% and 14.3% (low), respectively.

### Statistical Analyses

For all analyses, we considered only those days of observation for which ≥1 h of focal data was recorded and the male mate-guarded a female for ≥25 % of observation time. We discarded from the analyses the days during which the male did not mate-guard the same female for at least 70 % of his mate-guarding time, and days during which females where mate-guarded after conception (see Engelhardt *et al*. 2007; we discarded 12.5 % of the mate-guarding days, i.e., 23 of 215 d). We determined the likely date of conception for each new born in each group each year by counting back 163 d (mean gestation length in this study population; Engelhardt *et al*. 2006) from the date of birth of each newborn. We considered each mate-guarding day occurring >2 wk after the likely date of conception to be a post-conception day. We included all days for females that did not conceive in a given year because they may have been cycling at the time even if they did not conceive. The final data set comprised 733 h of focal observations over 192 d (details in Table I).

### Influence of Female Rank, Parity Status, and Male–Female AI on the Costs of Mate-Guarding

For each day, we calculated the percentage of time spent feeding, grooming, and being vigilant as percentage of the observation time for the focal male. In addition, we determined the average canopy height difference, i.e., vertical locomotion, per minute (in meters). We also calculated the copulation rate (number of copulations between the focal male and any female per hour), the rate of male–male aggression (the number of aggressions between the focal male and any other adult male per hour), the number of males in proximity (the mean number of males within 10 m per 5-min scan), and the number of sexually active females in each group on each observation day. We assessed male stress hormone levels (fGC measures) on days for which we had matching fecal samples. Because the time-lag for excretion of glucocorticoid metabolites into the feces is on average 36 h in long-tailed macaques (Heistermann *et al*. 2006), we matched behavioral observations with fGCs levels measured in samples collected at either day +1 or day +2 after the observations. When samples were available for both days, we used the mean fGCs levels of the two samples.

We used generalized linear mixed models (GLMM; Baayen 2008) to test whether the rank of the guarded female, her parity status and male–female AI had an effect on a male’s 1) feeding time (model 1), 2) climbing distance (model 2), and 3) fGC levels (model 3). The structure of each model is summarized in Table II. The fGC level values were log-transformed and climbing distances were power transformed (^0.7) to achieve a symmetric distribution. We used a Gaussian error structure in the models. Because fruit availability affects feeding time, distance climbed, and fGC levels in our study males (Girard-Buttoz 2014; Girard-Buttoz *et al*. 2014) we included fruit availability as control predictor in each model. Fruit availability on a given day was approximated using the fruit availability measured on the closest monthly record (details in Girard-Buttoz *et al*. 2014). We also included percentage daily mate-guarding time (as percentage of observation time) and number of females in the group as control predictors. In addition, in model 3, we also included variables that are known to affect fGC excretion in primates in general and/or in our population in particular as control predictors (Cheney & Seyfarth 2009; Girard-Buttoz *et al*. 2009; Girard-Buttoz 2014; Ray and Sapolsky 1992; Warm *et al*. 2008), i.e., male–male aggression rate, copulation rate, grooming time, number of male in proximity, the number of sexually active females, and the interaction between vigilance time and mate-guarding time.

**Table II:** Structure of models 1–6, study on male long-tailed macaques (*Macaca fascicularis*) at Ketambe, Gunung Leuser National Park, Indonesia (2010–2011)

	Model 1	Model 2	Model 3	Model 4	Model 5	Model 6
Response	Feeding time	Climbing distance (^0.7)	Log (fGC levels)	Aggression given (Y/N)	Distance to the female	Vigilance time
Fixed factors	Female rank Female parity status Male–female AI Male–female AI * fruit availability	Female rank Female parity status Male–female AI Male–female AI * fruit availability	Female rank Female parity status Male–female AI Female rank * female parity status	Female rank Female parity status Male–female AI	Female rank Female parity status Male–female AI Male–female AI * fruit availability Female parity * fruit availability	Female rank Female parity status Male–female AI Male–female AI * fruit availability
	*Fruit availability* *N females in the group* *Mate-guarding time* *Male rank*	*Fruit availability* *N females in the group* *Mate-guarding time* *Male rank*	*Fruit availability* *N females in the group* *Mate-guarding time* *Male rank* *N males in proximity* *N sexually active females* *Copulation rate* *Grooming time* *Vigilance time* Vigilance * mate-guarding time	*Fruit availability* *N females in the group* *Mate-guarding time* *Male rank* *N. males in proximity* *N. sexually active females*	*Fruit availability* *N females in the group*. *Mate-guarding time* *Male rank* *N males in proximity* *N sexually active females*	*Fruit availability* *N females in the group* *Mate-guarding time* *Male rank* *N males in proximity* *N sexually active females*
				*Observation time*		
Random factors	Male ID Guarded female ID group	Male ID Guarded female ID group	Male ID Guarded female ID group	Male ID Guarded female ID group	Male ID Guarded female ID group	Male ID Guarded female ID group

The control fixed factors are indicated in italics.

Male primates often prefer to mate with and/or mate-guard parous than nulliparous females regardless of their rank (Muller *et al*. 2006; Setchell 1999; Smuts 1985). The effect of female rank on the costs of mate-guarding may therefore be present only for parous females. To test for this eventuality we assessed the significance of the interaction between parity status and rank in all of the three models. This interaction was not significant in models 1 and 2 [likelihood ratio test (LRT), *P* > 0.1] but there was a trend toward significance in model 3 (LRT, *P* = 0.055).

To assess whether ecological factors modulate the relationship between costs of mate-guarding and female rank, parity status, and male–female AI, we also tested for the significance of the interaction between these three parameters and fruit availability in each model. The interaction between male–female AI and fruit availability was significant only in models 1 and 2 (LRT, *P* < 0.05).

### Influence of Female Rank, Parity Status, and Male–Female AI on the Investment of Males into Mate-Guarding

In a second set of models we assessed whether males are more motivated and engage more in costly behaviors when they mate-guard females of high reproductive value, i.e., high-ranking parous females, or with which they are closely bonded, i.e., high AI, than when mate-guarding other females. We used GLMMs to analyze the effect of female rank, parity status, and male–female AI on 1) likelihood of severely aggressing other males (model 4), 2) distance to the mate-guarded female (model 5), and 3) vigilance time (model 6). We calculated vigilance time using data collected during the focal time, i.e., both mate-guarding and non–mate-guarding instantaneous minute scan samples of each mate-guarding day, to provide an accurate representation of the male’s time budget that day. In contrast, the distance to the mate-guarded females is meaningful only during the instantaneous minute scans during which the males were mate-guarding and hence this distance was calculated only based on the mate-guarding minutes. The structure of each model is summarized in Table II.

Severe aggressions comprised chase, hit, and bite. The focal males did not severely aggress any male during over half of the observation days (101/192) and hence the resulting distribution of daily aggression given was highly zero inflated. Accordingly we could not run a model with a Gaussian error structure. We thus coded each day with at least one severe aggression given by the focal male toward any other male in the group as an “aggressing” day and other days as “non-aggressing” days. We used a model with a binomial error structure to test the influence of mate-guarding on the likelihood of aggression with other males on a given day (model 4). For the other models we used a Gaussian error structure because the response variable was symmetrically distributed. As the likelihood of recording severe aggression given on a given day was dependent on the observation time that day, we included observation time (in minutes) as a control predictor in model 4. In long-tailed macaques, fruit availability affects male locomotion (and hence also potentially the distance a male maintains with the female during mate-guarding), the trade-off between vigilance and feeding (Girard-Buttoz 2014) and potentially also the degree of male-male competition for access to food. Similar to models 1–3 we thus included this parameter as a control predictor in models 4–6. Finally, because the investment of males into mate-guarding can be modulated by sociosexual context and degree of male–male competition we also included number of sexually active females on a given day and number of males in proximity (see definition given earlier) as control predictors into these models.

In this set of models, we also tested for the significance of several interactions: 1) the interactions between fruit availability and female rank, parity status, and male–female AI to assess whether ecological factors modulate the relationship between these three parameters and male investment during mate-guarding and 2) the interaction between female rank and parity status for the same rational basis as in models 1–3. The interaction between male–female AI and fruit availability was significant in models 5 and 6 (LRT, both *P* < 0.05). All other LRT tests revealed *P* > 0.1.

For all models (models 1–6) all interactions with *P* < 0.1 for the LRT were kept in the final models. Finally, in addition of all the fixed factors mentioned previously, we included, in all models, male rank as a control fixed factor and male ID and group as nested random effects.

### Models Fitting and Assumptions Checking

Each model was fitted in R 2.15.0 (R Development Core Team 2012) using the function lmer of the R-package lme4 (Bates and Maechler 2010).

In each model, we checked that the assumptions of normally distributed and homogeneous residuals were fulfilled by visually inspecting a qqplot and the residuals plotted against fitted values. We checked for model stability by excluding data points one by one from the data and comparing the estimates derived with those obtained for the full model. In some models we identified influential cases that rendered the model unstable. We then reran the models without these particular data points. If the results were similar with or without influential cases we present the outcome of the model run on the full data set. If the results were different, we present the outcome of the models run with a reduced data set, i.e., not comprising influential cases. We derived variance inflation factors (VIF, Field 2005) using the function vif of the R-package car (Fox and Weisberg 2010) applied to a standard linear model excluding the random effects. VIFs <5 indicate that covariation between the predictors is not a problem (Bowerman and O’Connell 1990; Myers 1990). In all our models VIFs were <2.2. The other diagnostics did not indicate obvious violation of the assumption.

For each model, we first determined the significance of the full model as compared to the corresponding null model (including all the factors except “female rank,” “female parity status,” “male–female AI,” and the interactions) using a likelihood ratio test (R function anova with argument test set to Chisq). To achieve a more reliable *P*-value, we fitted the models using maximum likelihood rather than restricted maximum likelihood (Bolker *et al*. 2009). If this likelihood ratio test revealed *P* < 0.1 we considered the significance of the individual predictors. *P*-values for the individual effects were based on Markov Chain Monte Carlo sampling (Baayen 2008) and derived using the functions pvals.fnc and aovlmer.fnc of the R package languageR (Baayen 2010; number of simulations = 10,000).

## Results

### Mate-Guarding Duration, Female Value, and Male–Female AI

All males but one (α male of group KA) mate-guarded nulliparous and parous females and all males but one (β male in group KA) mate-guarded females ranging from high to low ranking (Table I). Our data set is thus not biased toward certain males mate-guarding exclusively females with certain characteristics, e.g., high-ranking males mate-guarding only high-ranking or parous females.

**Fig. 1:**
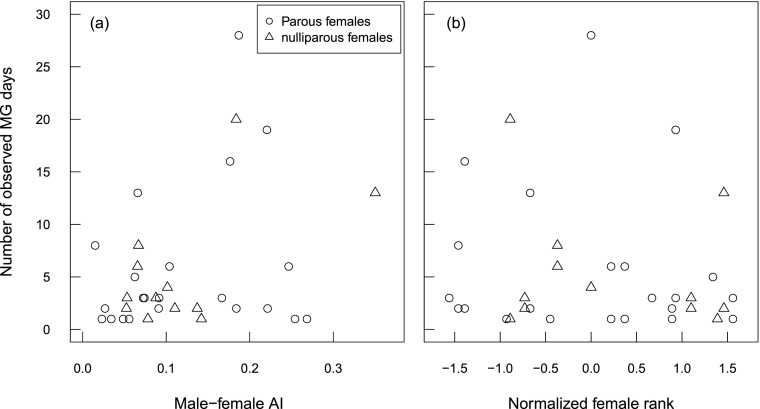
Number of days each female long-tailed macaque (*Macaca fascicularis*) was observed being mate-guarded by a given male depending on male–female association index **(a)** and female rank **(b)**, at Ketambe, Gunung Leuser National Park, Indonesia (2010–2011). Parous females are depicted with circles and nulliparous ones with triangles. Each point represents a given male-female guarding dyad.

The number of mate-guarding days was generally not related to female parity, rank, or male–female AI (Fig. 1). Whereas males spent more time on mate-guarding parous than nulliparous females in two groups (C and KA), the opposite occurred in the third group (KB) (Table I). Of all females that were observed to be mate-guarded, nulliparous females were guarded by a given male between 1 and 20 d and parous females between 1 and 28 d. Further, several females with an AI with the guarding male that was above the mean (mean AI = 0.121, range = 0.015–0.350) were mate-guarded only for 1 or 2 d, whereas several females with AI with the guarding male below the mean were guarded for >5 d or even for 14 d in the case of one female. Finally, a few low-ranking females were guarded for >10 d whereas many high-ranking females were mate-guarded for only 1 or 2 d.

### Influence of Female Rank, Parity Status, and Male–Female AI on the Costs of Mate-Guarding

#### Feeding Time (Model 1)

**Fig. 2:**
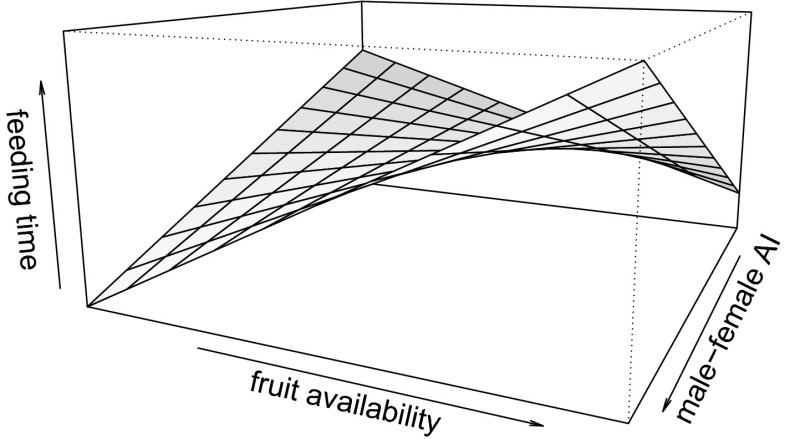
Effect of male–female Association Index (AI) and fruit availability on male long-tailed macaque (*Macaca fascicularis*) feeding time, at Ketambe, Gunung Leuser National Park, Indonesia (2010–2011). The plane depicts values predicted by model 1.

The full model was significantly different to the null model in model 1 (feeding time, *P* < 0.001, Table III). Males spent more time feeding when mate-guarding parous females than when mate-guarding nulliparous ones (*N* = 192 d, at reference level “parous” estimate ± SE = 5.73 ± 1.86, *P*_MCMC_ *=* 0.014, Table III), but female rank did not affect time spent feeding (*P*_MCMC_ *=* 0.794). A male’s AI with the guarded female influenced his feeding time through an interaction with fruit availability (*P*_MCMC_ *=* 0.001, Table III). When fruit availability was low, males fed less time while mate-guarding females with which they had a high AI. However, above a certain level of fruit availability, the pattern was reversed and males spent more time feeding while mate-guarding females with which they had a high AI (Fig. 2).

**Table III:** Results of the likelihood-ratio-tests (LRT) run to compare full vs. null models, estimates ± SE, *t*/*Z*-value, and *P*-values for the GLMMs run to test the influence (during mate-guarding) of male-female AI, female rank, and female parity status on male’s 1) feeding time (model 1), 2) fGC levels (model 3), 3) likelihood of aggressing other males (model 4), and 4) vigilance time (model 6), in long-tailed macaque (*Macaca fascicularis*) at Ketambe, Gunung Leuser National Park, Indonesia (2010–2011)

	Model 1 Feeding time			Model 2 Climbing distance			Model 3 fGC levels			Model 4 Aggression given			Model 6 Vigilance time		
No. of observation days	192			191			113			189			191		
Null vs. full model	*df*	χ^2^	*P*	*df*	χ^2^	*P*	*df*	χ^2^	*P*	*df*	χ^2^	*P*	*df*	χ^2^	*P*
	4	118.90	**< 0.001**	4	8.93	0.063	4	9.71	0.069	3	9.97	**0.019**	*4*	*9.88*	**0.042**
	*Estimate ± SE*	*t*	*P*_MCMC_	Estimate ± SE	*t*	*P*_MCMC_	Estimate ± SE	*t*	*P*_MCMC_	Estimate ± SE	*Z*	*P*	Estimate ± SE	*t*	*P*_MCMC_
Intercept	35.23 ± 1.48	23.77	**0.005**	1.02 ± 0.06	17.48	**0.003**	6.62 ± 0.09	72.30	**<0.001**	0.31 ± 0.46	0.72	0.470	43.93 ± 1.39	31.55	**0.001**
Male–female AI	In an interaction			In an interaction			–0.03 ± 0.05	–0.65	0.605	0.78 ± 0.28	2.83	**0.005**	In an interaction		
Female rank	0.35 ± 0.85	0.41	0.794	0.04 ± 0.03	1.37	0.218	In an interaction			–0.52 ± 0.23	–2.21	**0.027**	–2.10 ± 0.78	–2.69	**0.048**
Female parity status (parous)	5.73 ± 1.86	3.08	**0.014**	0.00 ± 0.07	0.05	0.959	In an interaction			–0.60 ± 0.51	–1.16	0.246	0.20 ± 1.76	0.12	0.716
Female rank * female parity							–0.21 ± 0.10	–2.05	0.072						
Assoc. index * fruit availability	3.25 ± 0.82	3.96	**0.001**	0.06 ± 0.02	2.60	**0.012**							–1.62 ± 0.75	–2.17	0.155
Fruit availability	In an interaction			In an interaction			–0.25 ± 0.05	–5.46	**<0.001**	–0.34 ± 0.25	–1.37	0.170	In an interaction		
Number of females	–3.22 ± 0.96	–3.35	0.131	–0.12 ± 0.04	–3.32	**0.013**	0.00 ± 0.06	0.06	0.781	0.16 ± 0.21	0.76	0.448	0.80 ± 0.99	0.81	0.504
MG time	0.35 ± 0.85	0.41	0.633	–0.05 ± 0.02	–2.01	0.065	In an interaction			0.19 ± 0.21	0.92	0.359	1.15 ± 0.78	1.49	0.331
Male rank (β)	–6.06 ± 1.79	–3.39	0.056	–0.1 ± 0.06	–1.82	0.182	–0.31 ± 0.09	–3.38	0.069	0.65 ± 0.46	1.40	0.160			
No. of males in proximity							–0.09 ± 0.04	–2.07	0.087	1.21 ± 0.27	4.46	**<0.001**	–1.52 ± 0.82	–1.87	0.104
No. of sexually active females							0.05 ± 0.04	1.21	0.218	0.16 ± 0.21	0.76	0.448	–1.01 ± 0.79	–1.28	0.213
Copulation rate							0.01 ± 0.04	0.33	0.753						
Grooming time							0.00 ± 0.04	0.00	0.851						
Observation time										1.57 ± 0.33	4.74	**<0.001**			
Vigilance time							In an interaction								
MG time * vigilance							–0.03 ± 0.04	–0.64	0.586						

In this table only the models for which LRT to compare null vs. full models revealed *P* < 0.1 are presented.

*P* values <0.05 are indicated in bold

#### Climbing Distance (Model 2)

**Fig. 3:**
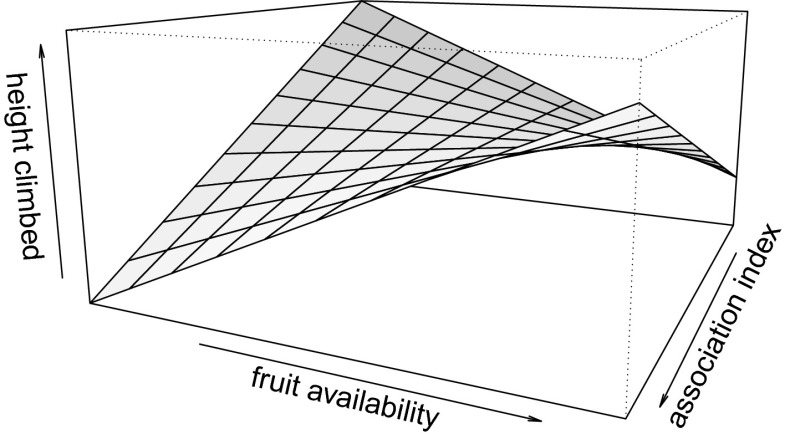
Effect of male–female Association Index (AI) and fruit availability on male long-tailed macaque (*Macaca fascicularis*) climbing distance, at Ketambe, Gunung Leuser National Park, Indonesia (2010–2011). The plane depicts values predicted by model 2.

In model 2, the comparison of the null and the full model revealed a trend toward significance (*P* = 0.063, Table III). Similar to model 1, a male’s AI with the guarded female influenced his distance climbed through an interaction with fruit availability (*P*_MCMC_ = 0.012, Table III). When fruit availability was low, males climbed less distance while mate-guarding females with which they had a high AI. However, above a certain level of fruit availability, the pattern was reversed and males climbed more distance while mate-guarding females with which they had a higher AI (Fig. 3).

#### fGC Levels (Model 3)

**Fig. 4:**
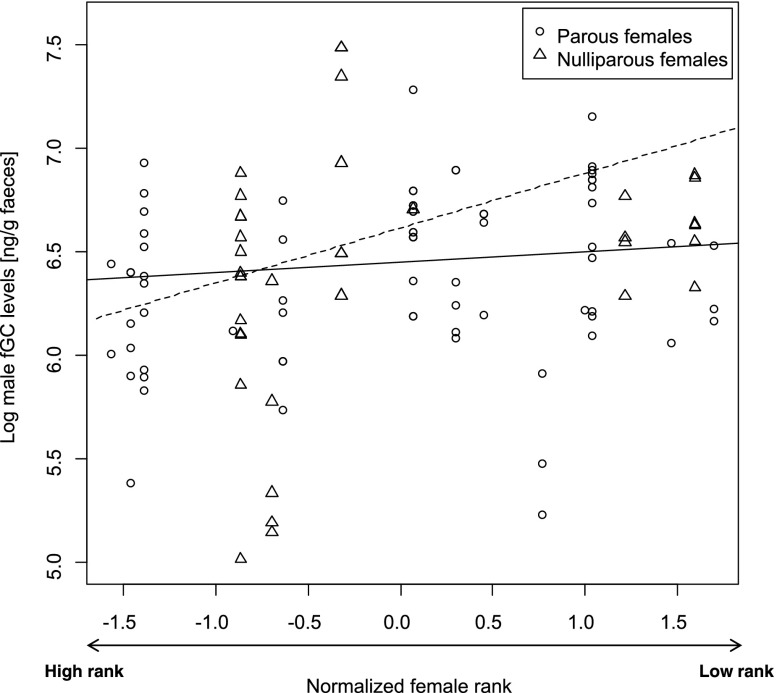
Effect of the guarded female’s rank on male long-tailed macaque (*Macaca fascicularis*) fGC levels for nulliparous females (triangles) and parous females (circles), at Ketambe, Gunung Leuser National Park, Indonesia (2010–2011). The lines depict the linear relationship predicted by model 3 for nulliparous (dashed line) and parous females (solid line) and the dots depict the raw data points.

In model 3, the comparison between the null and the full model revealed a trend toward significance (*P* = 0.069, Table III). The AI between the male and the guarded female did not influence male’s fGC levels (*N* = 113 d, *P* = 0.605). The interaction between “female rank” and “parity status” was close to significance (*P* = 0.072) and males tended to have higher stress hormone levels when mate-guarding low-ranking nulliparous females than when mate-guarding high-ranking nulliparous females (Fig. 4). Finally fruit availability had a significant and negative effect on a male’s fGC levels (estimate ± SE = –0.25 ± 0.05, *P*_MCMC_ < 0.001, Table III). The more fruits were available the lower were the males’ fGC levels.

### Influence of Female Rank, Parity Status, and Male–Female AI on Male Investment During Mate-Guarding

#### Aggression Given (Model 4)

The full model was significantly different from the null model in model 4 (*P* = 0.019, Table III). The likelihood of aggressing other males was affected significantly by male–female AI (*N* = 189 d, estimate ± SE = 0.78 ± 0.28, *P*_MCMC_ *=* 0.005, Table III) and female rank (estimate ± SE = –0.52 ± 0.23, *P*_MCMC_ *=* 0.027, Table III) but not by female parity status (*P*_MCMC_ = 0.246). Males were more likely to aggress other males when mate-guarding high-ranking females and those with which they had a high AI.

#### Distance to the Female (Model 5)

In model 5, the full model was not significantly different from the null model (LRT, df = 5, χ^2^ = 9.09, *P* = 0.105), indicating that neither male–female AI nor female rank or parity status significantly affected the distance males maintained with the female during mate-guarding.

#### Vigilance Time (Model 6)

In model 6, the full model was significantly different from the null model (LRT, df = 4, χ^2^ = 9.88, *P* = 0.042). A male’s vigilance time during mate-guarding was significantly affected by female rank (*N* = 191, estimate ± SE = –2.10 ± 0.78, *P*_MCMC_ *=* 0.048, Table III) but not by female parity status or male–female AI (both *P* > 0.15, Table III). Males were more vigilant when mate-guarding high-ranking females than when mate-guarding low-ranking ones.

## Discussion

Our results indicate that female rank, parity, and the strength of male–female social bonds affect behavioral and physiological costs of mate-guarding and male investment in this behavior in wild long-tailed macaques. Specifically, our results suggest that females of lower reproductive value, i.e., nulliparous and low-ranking females, might be more costly to mate-guard than females of high reproductive value. Males spent less time feeding when mate-guarding nulliparous than parous females and tended to be more physiologically stressed when mate-guarding low-ranking nulliparous females than other females. Further, the male–female social bond strength (AI) influenced a male’s feeding time during mate-guarding but this effect was contingent on fruit availability. When fruit availability was high, males fed longer while mate-guarding females with which they were strongly bonded than while mate-guarding other females; this pattern was reversed when fruit availability was low. Finally, males appear to invest more in females of high reproductive value or in females with which they are strongly bonded than in other females. Males were more vigilant and aggressive when mate-guarding high-ranking and/or females with which they were strongly bonded (high AI) than when mate-guarding other females.

Feeding and stress-related costs of mate-guarding have been reported in different primate species, including long-tailed macaques (Alberts *et al*. 1996; Bergman *et al*. 2005; Girard-Buttoz 2014; Girard-Buttoz *et al*. 2014; Matsubara 2003; Packer 1979; Rasmussen 1985). Our study shows that male long-tailed macaques may have the opportunity to limit these costs by mate-guarding preferentially parous high-ranking females. For male long-tailed macaques the cost/benefit ratio might be higher when mate-guarding low-ranking nulliparous females because the costs are higher (lower food intake and higher physiological stress) and the benefits are lower (lower quality offspring) than when mate-guarding high-ranking parous females. First, in our study, males fed for longer when mate-guarding parous than nulliparous females. Males may thus benefit from preferentially mate-guarding parous females in terms of balancing their energetic status. Further, despite the absence of an effect of female rank on male feeding time, males may also benefit from mate-guarding high-ranking females. Whereas male long-tailed macaques trade-off vigilance time against feeding time during mate-guarding in general (Girard-Buttoz 2014), this might be the case less when they mate-guard high-ranking females than when they mate-guard low-ranking females. In fact, males were more vigilant when mate-guarding high-ranking females than when mate-guarding low-ranking ones but achieved this without reducing their feeding time. The absence/reduction of the trade-off between feeding and vigilance when mate-guarding females of high rank might be related to the fact that high-ranking females have priority of access to high-quality food patches (van Noordwijk and van Schaik 1987) from which mate-guarding males may also benefit. Consequently, males may mate-guard females of high reproductive value more thoroughly than females of low reproductive value by being more vigilant without paying extra costs of reduced feeding time.

Second, in our study, males were more physiologically stressed when mate-guarding low-ranking nulliparous than high-ranking parous females. This result is somewhat surprising because we might expect that higher male–male competition for females with the highest reproductive value, i.e., parous, high-ranking females, would be stressful for the mate-guarding male. However, this may not have been the case in our study males because they were rarely challenged during mate-guarding (Girard-Buttoz 2014). Further, male–male aggression did not affect male stress levels significantly in a previous study on long-tailed macaques (Girard-Buttoz 2014). In contrast, female spatial positioning and centrality in the group may explain the effect of rank and parity on male physiological stress levels. In long-tailed macaques, low-ranking females spend usually more time in the periphery of the group than high-ranking females (van Noordwijk and van Schaik 1987). When mate-guarding low-ranking females, males may therefore also occupy a less central position in the group than when mate-guarding high-ranking females. In turn, not being at the core of the group is likely to increase a male’s stress levels for several reasons. First, our study males are more stressed when they have fewer males in proximity (Girard-Buttoz 2014) and hence fewer potential allies to support them and form coalitions against extra-group male consort or rank takeover attempts (van Noordwijk and van Schaik 2001). Second, being at the periphery of the group may enhance the risk of exposure to predators (Ron *et al*. 1996), which may be stressful. Third, a less central spatial position reduces the opportunity of interacting with other females and sociosexual interactions with females may be of prime importance for males in this species. In long-tailed macaques, it has been suggested that males need to copulate with females to monitor the timing of their fertile phase because female sexual behavior but not sex skin swelling reliably indicates the female fertile window (Engelhardt *et al*. 2005). While mate-guarding a low-ranking nulliparous female, males may thus be stressed because they may not be able to monitor the fertility status of other females and hence lose the opportunity to fertilize females of higher reproductive value. Yet whether male long-tailed macaques have the cognitive ability to track the fertility status of several females simultaneously while mate-guarding remains to be investigated.

Our data suggest that the males may lower the risk of exposure to repeated elevation of physiological stress by preferentially mate-guarding high-ranking, parous females compared to low-ranking nulliparous ones. This might be particularly important in a non-strictly seasonal species, such as long-tailed macaques, in which an increase in stress levels during mate-guarding (Girard-Buttoz 2014) might have deleterious consequences. In this species, the timing of female fertility periods is unpredictable and females can conceive year round (van Schaik and van Noordwijk 1985). Further, female fertile phases are usually asynchronous (Engelhardt *et al*. 2006). Therefore, by mate-guarding one female after the other, males may be exposed to repeated elevations in their glucocorticoid levels and eventually face the risk of becoming chronically stressed (Girard-Buttoz 2014). This might be highly costly for the males because chronic stress may suppress the immune system (Grossman 1985; Setchell *et al*. 2010) and testicular function (Hardy *et al*. 2005; Sapolsky 1985) and hence affect males’ health and ability to reproduce (*cf.* Boonstra 2013).

In long-tailed macaques and other primate species, low-ranking and/or nulliparous females produce offspring that are less likely to survive until adulthood and to achieve a high rank position in the future than offspring of high-ranking and/or parous females (Bercovitch *et al*. 1998; Majolo *et al*. 2012; Robbins *et al*. 2006, 2011; Setchell *et al*. 2002; van Noordwijk and van Schaik 1999, 2001). Evolutionary pressures at both proximate and ultimate levels may therefore bias male mate-guarding choice toward high-ranking parous females which appear to be less costly to monopolize and are likely to achieve higher reproductive success. Given that high-ranking males usually have priority of access to females (Berard *et al*. 1994; Bercovitch 1997; de Ruiter *et al*. 1994; Engelhardt *et al*. 2006; Matsubara 2003; Setchell *et al*. 2005) they are less constrained in their mate-guarding choices than other males. High -ranking males may therefore benefit from their dominance status twice over: by being able to access and mate-guard females with high reproductive value successfully and by reducing the costs of female monopolisation while doing so. In view of this, it is surprising that in our study males did not obviously choose females of high reproductive value as preferred mate-guarding partners. This pattern contrasts, at least partially, with results from a previous study of the same population (Engelhardt *et al*. 2006) that found that higher ranking females were mate-guarded longer by the α male during their fertile phase than lower ranking females. In the study by Engelhardt and colleagues (2006), female fertile phases did not overlap and the focal group comprised only 8 females. Our study groups had up to 15 females and thus the likelihood of temporal overlap of fertile phases is much greater. However, we did not have the logistical power to collect regular fecal samples from all the females of all the study groups so as to be able to assess temporal overlap of female fertile phases. We cannot, therefore, draw definite conclusions regarding male mate-guarding choice. For example, it may well be that when two females are fertile at the same time high-ranking males mate-guard high-ranking or parous females preferentially.

Beyond the time spent mate-guarding different females, the selectivity of males toward certain females may also be expressed at the level of the investment and thoroughness with which males mate-guard the females. We found that males were more aggressive and more vigilant when mate-guarding high-ranking females than when mate-guarding low-ranking ones. These two parameters may reflect an active decision of the males to enhance the efficiency of monopolizing females of high rank and thus higher reproductive value. In this respect, our focal males follow patterns described in long-tailed macaques and other species of primates with male mating and/or mate-guarding preference toward higher-ranking females (de Ruiter *et al*. 1994; Engelhardt *et al*. 2006; Kuester and Paul 1996; Setchell and Wickings 2006). By aggressing other males, the mate-guarding male may face counter-aggressions and is thus exposed to a higher risk of injuries (Blanchard *et al*. 1988; Clutton-Brock *et al*. 1979; Drews 1996). Being injured may, in turn, prevent high-ranking males from mate-guarding current or subsequent fertile females and/or maintaining their hierarchical status (Drews 1996; C. Girard-Buttoz *pers obs*). Given the strong link between rank and reproductive success in male long-tailed macaques (de Ruiter *et al*. 1994; Engelhardt *et al*. 2006) males may only be willing to face the risk of aggressive retaliation, and the likelihood of associated deleterious consequences, to monopolize females of high reproductive value.

Beyond enhancing the efficiency of monopolization, being more vigilant while mate-guarding high-ranking females may also partially modify male physiological stress levels. In a previous study we found that males had lower physiological stress levels the more vigilant they were during mate-guarding (Girard-Buttoz 2014). This may explain our finding of lower glucocorticoid levels when males were mate-guarding high-ranking compared to low-ranking nulliparous females.

In addition to female rank and parity status, male–female social bonds also affected costs of mate-guarding and male investment in this behavior. The strength of male–female social bonds affected the time spent feeding and the climbing distance of the mate-guarding male, although this effect was contingent on fruit availability. In periods of high fruit availability, males fed more but also climbed more when the association index with the guarded female was higher. In this context, increased feeding time may stem from greater cooperation by the female. It has, in fact, been suggested and reported in some species that male–female social bonds influence female cooperation during mate-guarding in primates (Rasmussen 1980; Smuts 1985; *cf.* Manson 1994). In turn, feeding for longer may imply the exploitation of more food patch and hence increased locomotion (Alberts *et al*. 1996), which may explain why increased feeding time was accompanied by an increase in vertical distance climbed in our focal males. In contrast, when fruit availability was low, males fed less and climbed less when mate-guarding females with which they were strongly bonded. This may indicate a higher degree of investment of the males into mate-guarding strongly bonded females. In the context of fruit scarcity, males may face a trade-off between feeding long enough to meet their energetic requirement and being able to mate-guard females thoroughly. Under such conditions, males may therefore only thoroughly mate-guard the female which they are strongly bonded at the costs of reduced feeding. In contrast, if the female is not strongly bonded to him, the male may favour energetic needs over mate-guarding investment and relax mate-guarding attention to feed longer. However, because feeding time and climbing distance covary, a potential decrease in males’ energy intake (related to lowered feeding duration) may be counterbalanced by a decrease in male energy expenditure (associated with a decreased distance climbed see Girard-Buttoz 2014). The effect of male–female social bond strength on male energetics remains therefore to be shown, using physiological markers of energetic status such as urinary C-peptide (Deschner *et al*. 2008; Emery Thompson and Knott 2008; Emery Thompson *et al*. 2009; Girard-Buttoz *et al*. 2011; Higham *et al*. 2011; Sherry and Ellison 2007).

The fact that males were also more aggressive when mate-guarding females with which they are strongly bonded further shows their increased investment in these females. Mate-guarding females more thoroughly and preventing other males from accessing them more aggressively might be a mechanism for the mate-guarding male to maintain strong social bonds with certain females. In turn, this bond may have direct fitness benefit for the males, as shown in rhesus macaques (Kulik *et al*. 2012; Massen *et al*. 2012). In long-tailed macaques, females are not fully monopolizable because females can gain copulations via non–mate-guarding males (de Ruiter *et al*. 1994). Accordingly, cryptic post-copulatory female choice has been hypothesized to play a role in offspring paternity (Engelhardt *et al*. 2006; see Kappeler 2012 for possible mechanisms). It may, therefore, be of high importance for males, even high-ranking ones, to maintain strong social bonds with certain females to enhance their probability of post-copulatory sperm selection.

Our study brings a new dimension to the study of mate choice in primates (Kappeler 2012; Keddy-Hector 1992; Setchell and Kappeler 2003) by showing that males might be constrained in their mate-guarding choices by both social and ecological factors. Further, we showed that beyond differential time allocation of mate-guarding (Engelhardt *et al*. 2006; Setchell and Wickings 2006), males may express preference toward highly valuable females (those with high reproductive potential or those to which they are closely bonded) by investing more in aggression and vigilance while mate-guarding these females. A male may thus not only mate preferentially with the most valuable females (Kappeler 2012; Setchell and Kappeler 2003) but also aim to securing paternity better through optimized monopolization (our study). Our findings support mathematical modeling and evolutionary theories (Edward and Chapman 2011; Kokko and Monaghan 2001) predicting that when females vary in quality (van Noordwijk and van Schaik 1999, 2001) and the access to and monopolization of females is costly (Girard-Buttoz 2014), males may express choosiness toward certain females. Future studies should investigate how, at a given point in time, males adjust their mating and/or mate-guarding decisions depending on the interplay between their physiological and physical conditions, the food available, and the quality and diversity of females in their fertile phase. Such studies will require hormonal assessment of female fertile phase (Dubuc *et al*. 2012; Engelhardt *et al*. 2004; Heistermann *et al*. 2001; Young *et al*. 2013) but also the use of noninvasive physiological markers of male body condition and energetic status, such as urinary C-peptides (Deschner *et al*. 2008; Emery Thompson and Knott 2008; Emery Thompson *et al*. 2009; Girard-Buttoz *et al*. 2011; Higham *et al*. 2011; Sherry and Ellison 2007) or stable Isotopes (Deschner *et al*. 2012).
